# Supplementary material for: Assessing the effectiveness of no-take zones on fish populations in the Marine Natural Park of Cap Corse and Agriate, Northwestern Mediterranean Sea
Source: PLoS One. 2025 Sep 2;20(9):e0327641. doi: 10.1371/journal.pone.0327641 (PMC12404554; doi:10.1371/journal.pone.0327641)
Supplement: Attachment — Submitted filename: Rebuttal_Letter_PlosOne.pdf [file pone.0327641.s001.pdf]

Rebuttal Letter – Manuscript PONE-D-24-44762 – Assessing the effectiveness of no-take zones on fish populations in the Marine Natural Park of Cap Corse and Agriate, Northwestern Mediterranean Sea

Dr. Lucie Vanalderweireldt

April 25, 2025

Dear Academic Editor and Reviewers,

Thank you for the time you invested in evaluating our manuscript and for the constructive comments that helped us improve it. In response we have:

1. clarified the field methodology, giving particular attention to the two UVC survey designs;
2. added a new figure (Fig. 2) that illustrates the protocols and shows the three focal species;
3. refined the statistical analysis: we replaced quasi-Poisson models with log-transformed Gaussian GLMs, which yielded markedly better residual diagnostics and more transparent effect sizes;
4. extended the models to include biomass ($g \cdot 1000\ m^2$) in addition to numerical density.

These changes have strengthened both the presentation and the interpretation of our results. We trust that the revised manuscript addresses all points raised and look forward to your further feedback.

Editor's Comments:

Comment 1: I agree with the reviewers that the manuscript needs some revision to further improve it. I also suggest the authors add some photos of the targeted fishes in the study along with some scheme of the method described. I also suggest the authors to add, if possible, some raw footage of the methods as supplementary material.

Response 1: We have now taken steps to address this issue in our revised manuscript. We have added a new schematic figure illustrating the cross and comb approaches also including photos of the three target fish species in the main text. Additionally, we have expanded our Methods section to provide more detail and clarity on the two underwater visual census (UVC) protocols. We hope these additions will further enhance the manuscript's clarity and completeness.

Comment 2: We note that Figure 1 in your submission contain [map/satellite] images which may be copyrighted. All PLOS content is published under the Creative Commons Attribution License (CC BY 4.0), which means that the manuscript, images, and Supporting Information files will be freely available online, and any third party is permitted to access, download, copy, distribute, and use these materials in any way, even commercially, with proper attribution. For these reasons, we cannot publish previously copyrighted maps or satellite images created using proprietary data, such as Google software (Google Maps, Street View,

and Earth). For more information, see our copyright guidelines: http://journals.plos.org/plosone/s/licenses-and-copyright.

Response 2: We confirm that Figure 1 does not contain any copyrighted or proprietary imagery. We created the figure using QGIS and a habitat layer from Andromède Océanologie ("La Méditerranée dévoile ses dessous – Cartographie continue des habitats marins," 2014), which is freely accessible on the medtrix.fr platform. As such, the dataset is open access and is not subject to any copyright restrictions. We have ensured that all underlying data and the final figure comply with the CC BY 4.0 license requirements.

Reviewers' Comments and Responses:

Reviewer 1:

Comment 3: I have reviewed the manuscript titled "Assessing the effectiveness of no-take zones on fish populations in the Marine Natural Park of Cap Corse and Agriate, Northwestern Mediterranean Sea," submitted to PLOS One (PONE-D-24-44762). While the work is interesting, it does not significantly contribute new knowledge to the field of marine protected area (MPA) science. The paper's focus aligns with numerous studies conducted globally on MPA effects. Unfortunately, the manuscript fails to advance our understanding of MPAs by introducing novel methods or findings. The authors concentrate on three apex predators as bioindicators in Mediterranean rocky shores. However, the Methodology section lacks a clear description of the sampling design, raising questions about the study's approach. The division into three sectors (Agriate, Canelle, and Nonza), with varying numbers of sampling points per sector, necessitates further explanation. A detailed description of the sampling protocol would enhance the article's clarity and understanding. The explanation of the Underwater Visual Census (UVC) methodology is insufficient and difficult to comprehend. The use of two distinct protocols, with varying numbers of observers, raises concerns about potential biases in data collection. While the intent to employ diverse methods is understandable, the unequal sampling effort across methodologies and sectors compromises the data's credibility and overall interpretation. I recommend a thorough revision of the manuscript, including a reanalysis of the data, before considering it for publication. In its current form, the article is primarily of local or regional interest and may be more suitable for a journal with a narrower geographic focus.

Response 3: We appreciate the reviewer's point that our study may appear more regional in scope and thus not significantly novel from a global perspective on MPAs. However, our aim here is twofold: 1) Contribute a foundational dataset on three emblematic Mediterranean fish species (*Dentex dentex*, *Epinephelus marginatus*, and *Sciaena umbra*). These species are widely recognized as indicators of ecological health and attract conservation interest throughout the region. By sharing our data and findings now, we hope to establish a dialogue and foster collaborations that could lead to broader-scale syntheses in the future—rather than waiting years to publish until a multi-nation data set is assembled. We believe that timely dissemination of these results is valuable not only to local management but also for informing ongoing MPA discussions in the wider Mediterranean. 2) Clarify and improve our methodology in response to the reviewer's concerns. We have revised the Methods section to provide greater detail on how each sector was surveyed, including precise sampling effort (transects per site) and how differences in diver teams (cross vs. comb protocols) are normalized by surveying a fixed area (1,000 m2 for cross transects). While more divers might imply a more extensive effort, our findings show that this does not always yield higher detection, especially for *E. marginatus*. We also emphasize that the overall sampling effort has remained consistent among Agriate, Canelle, and Nonza since the study's inception, with the only gap occurring in one Agriate survey when weather conditions prevented diving. If further clarifications are needed, we are happy to provide additional details.

In short, though this manuscript may not represent a "large-scale" MPA meta-analysis, we consider it "useful science" for managers and researchers working directly with these iconic Mediterranean fish species. These data establish a baseline that can be built upon by future collaborations across multiple regions and time frames.

Comments 4: Line 25: While the authors cite generalist papers, a relatively large number of studies have specifically examined MPA effects on these species: Harmelin-Vivien et al. 2008; Hackradt et al. 2014; Rojo et al. 2020, including outside the Mediterranean (Anderson et al. 2014 for *E. marginatus*).

Response 4: We have now added the references.

Comments 5: Line 27: Similarly.

Response 5: We have now added the references.

Comments 6: Line 28: It is true that "assessing the population status of these three bioindicator species provides valuable ecosystem insights," but the manuscript should address new information on this issue.

Response 6: We agree that simply emphasizing the importance of bioindicator species without providing new insights is insufficient. Our study goes beyond this by offering two key contributions: 1) Demonstrating NTZ Effectiveness: We show that the Nonza NTZ significantly benefits the population density and biomass estimates, underscoring the importance of well-enforced no-take zones within larger, multi-use marine parks. 2) Comparing UVC Protocols: We evaluate two distinct underwater visual census (UVC) protocols—the cross and comb methods—and identify the cross protocol as more effective for detecting *E. marginatus* and *S. umbra* in complex reef environments. This finding highlights the need for tailored monitoring methods based on species-specific behaviors and habitats. We have enhanced the Discussion and Conclusion sections to better articulate these contributions and their implications for marine conservation and monitoring strategies.

Comments 7: Line 35: Please provide reference for this citation.

Response 7: We have now added the following reference to support this statement: *Direction de la Mer et du Littoral Corse (2023). Arrêté n° R20-2023-03-10-00001 portant sur la réglementation de la pêche du denti ou du denti commun (Dentex dentex) en Corse. Ajaccio, France.* This official decree provides the regulatory framework regarding the fishing of *Dentex dentex* in Corsica.

Comments 8: Line 43: This is not entirely accurate when considering the impact of fisheries. Some studies have found that indirect human activities, such as recreational diving, may not significantly impact fish populations, especially groupers (Pereñíguez et al. 2023). This statement requires rephrasing.

Response 8: Thank you for pointing this out. We agree that recent studies (e.g., Pereñíguez et al. 2023) suggest that scuba diving, when properly managed, may not significantly impact fish populations like groupers. However, in our case, the Nonza-San Fiurenzu No-Take Zone has prohibited all forms of fishing and diving, primarily to ensure minimal human disturbance within this highly protected area. We have revised the sentence.

Comments 9: Line 52: However, the specific UVC method employed can directly influence the assessment of MPA effects on grouper, *Dentex dentex* and *Sciaena umbra* populations (see Rojo et al 2021)

Response 9: We appreciate the reviewer's reference to the study by Rojo et al. (2021), which underscores how census design, such as transect width or length, can affect the detectability of certain fish species, particularly large predators. However, our study differs in several key aspects that are important to consider:

Species Focus: While Rojo et al. monitored seven species, our study focuses on three emblematic species: Dentex dentex, Epinephelus marginatus, and Sciaena umbra. This targeted approach allows for a more in-depth analysis of these specific species, which are of particular conservation interest in the Mediterranean. Additionally, Rojo et al. (2021) did not discuss their results at the species level.

Effort and Methodology: Our study employs two distinct UVC protocols—the cross and comb methods—each with different levels of effort and potential disturbance. The comb protocol, involving up to eight divers and two boats, covers a larger area but may introduce more disturbance. In contrast, the cross protocol uses only two divers and one boat, potentially reducing environmental impact.

Specific Objectives: Our primary objective is to assess the effectiveness of the No-Take Zone (NTZ) within the marine park and to compare the two UVC protocols in terms of their ability to accurately monitor these

species. We aim to provide recommendations for selecting the most appropriate UVC method for future monitoring efforts, considering both accuracy and practical feasibility.

Therefore, although our scope is more focused (fewer species and different transect designs), our overall conclusion aligns with Rojo et al. (2021) in that UVC methods tailored to species behavior can yield more robust results for elusive top predators. However, our present study also highlights that increased sampling effort (e.g., more divers, larger teams) does not always guarantee more accurate detection, particularly when shy species like E. marginatus may be undercounted by a larger group of divers. In contrast, the smaller cross protocol in our design produced similar or higher counts for E. marginatus, underscoring the role of species-specific behaviors.

Thus, while we recognize the merits of larger roaming transects and distance sampling (as recommended by Rojo et al. 2021), our findings add that logistical simplicity—or smaller teams—can sometimes match or outperform more elaborate designs, depending on the target species' ecology and the monitoring goals, especially at a newly established marine park level, where enforcement capacity is a real issue.

Reference: Rojo et al. (2021). Detection of protection benefits for predatory fishes depends on census methodology. Aquatic Conservation: Marine and Freshwater Ecosystems 31: 1670-1685.

Comments 10: Line 51 to 64: I suggest removing this paragraph. The discussion of UVC methodology is outdated and does not significantly enhance the manuscript. Instead, a focused discussion on the ecological context of *S. umbra*, *E. marginatus*, and *D. dentex* in France/Corsica would be more engaging for readers.

Response 10: Could you please clarify whether the suggestion is to remove all UVC methodology discussions along the manuscript? or just this paragraph within the introduction?

Comments 11: Line 70 to 77: This section appears to be more appropriate for the Materials and Methods section. Please reorganize the text accordingly.

Response 11: We understand the importance of maintaining a clear separation between the Introduction and the Materials and Methods sections. However, in many manuscripts, it is standard practice to provide a brief overview of the methodology at the end of the Introduction to help readers understand how the research questions align with the chosen methods. Nevertheless, I have revised this paragraph to better align with the reviewer's expectations.

Comments 12: Line 91 to 94: If Site A differs from the others, a clear justification for its inclusion is necessary. The rationale for its selection is unclear. Additionally, if evidence of differences between Site A and the NTZs exists, the authors should provide a convincing argument that these differences are primarily due to protection levels rather than habitat-mediated factors.

Response 12: We acknowledge that our wording in lines 91–94 may have given the impression that Site A differs markedly from the other sites. In fact, we deliberately selected sites with comparable reef characteristics (i.e., rocky bottoms at 15–25 m depth) to minimize habitat-related variation and better isolate the effect of protection levels. While there is some coverage of *Posidonia oceanica* meadows in the Agriate sector, preliminary analyses (including Kruskal–Wallis tests, MANOVA, PERMANOVA, and GLM models) showed no significant effect of habitat type between sectors and on the density of the studied species. Hence, habitat differences are not driving the observed patterns, and we are confident that any differences between sites are primarily related to protection status rather than habitat-mediated factors. We have now modified the study site subsection accordingly, as well as added few sentences on the matter in the data analysis subsection.

Comments 13: Line 137: The rationale for testing survey time, SST, and chlorophyll concentration is questionable. It is unlikely that top predators like groupers are significantly influenced by chlorophyll concentration, especially considering their relatively small home ranges.

Response 13: We appreciate your feedback regarding the inclusion of survey time, SST, and chl-*a* concentration. Although groupers do have relatively small home ranges, we tested these variables to eliminate the possibility that any observed differences in their densities were driven by environmental or habitat-linked

factors rather than by protection status. SST and chl-a can serve as proxies for temperature regimes and local productivity, both of which may indirectly affect prey availability or habitat conditions. By including these parameters, we aimed to ensure that any observed reserve effect could be more confidently attributed to protection measures, rather than confounding environmental gradients.

Comments 14: Line 141: This corroborates my concern that the environmental data resolution the authors applied is biger than the fine-scale effects measurable by UVC. Including these variables in the GLM may introduce unnecessary variability, making it difficult to interpret the results. The authors may have overfitted the model by including excessive variables to explain the observed patterns, potentially leading to spurious correlations.

Response 14: Thank you for raising this concern. While the grid resolution may seem large, our primary goal in including these environmental variables was to ensure that any observed differences in fish densities were not confounded by environmental gradients. We verified that the resolution is sufficient to capture habitat contrasts between sectors (distances ranging from 7.3 up to 18.3 km), which aligns with the scale at which we observe local habitat or productivity differences. Including SST and chlorophyll-a in the GLMs thus allows us to better distinguish the effect of protection status from potential habitat-mediated effects.

Comments 15: Line 171: This analysis is rather weak. A mixed-effects model, which can handle unbalanced designs and non-normal data, would be a more suitable approach. GLMMs with Gaussian distributions can correct for the bias introduced by the unbalanced design. Alternatively, PERMANOVA could be considered, as it is more robust to non-normal data and reduces the risk of Type II errors. Additionally, analyzing biomass, which incorporates both size and abundance, could provide a more comprehensive assessment.

Response 15: We agree that biomass offers a more comprehensive assessment of fish assemblages by integrating both abundance and body size. In response, we expanded our analysis to include biomass density (g/1000 m^2) alongside population density and revised the results and discussion sections accordingly.

Biomass was estimated using species-specific length–weight relationships of the form $W = a \times L^b$, where L is the midpoint of each observed length class and coefficients a and b were obtained from FishBase. For each transect, total biomass was calculated as the sum over all size classes of the estimated weight multiplied by the number of individuals in each class, scaled by the survey area, and log-transformed using $\log(x+1)$ to stabilize variance.

We also revisited our statistical approach to improve model performance and interpretability. Earlier versions of the analysis used quasi-Poisson GLMs to account for overdispersion in count data, but we found that log-transforming the response variable and applying Gaussian GLMs led to substantially improved residual diagnostics which is why we chose to proceed with this approach.

In line with the reviewer's suggestion, we explored generalized linear mixed models (GLMMs) with a random intercept for UVC protocol to account for the unbalanced design. However, these models showed poor fit and frequent convergence issues. Furthermore, since we were specifically interested in evaluating the influence of the protocol itself, we chose to include it as a fixed effect in our final Gaussian GLMs. This allowed us to directly assess its effect rather than treating it as a source of random variation.

All final models included the same set of fixed effects: protection status, year, season, sea surface temperature (SST, in °C), Chlorophyll-a concentration (mg/m^3), and UVC protocol. Full results are presented for both log-transformed population density and biomass density models.

Comments 16: Line 228 to 234: I am not convinced by the proposed relationship between SST and chlorophyll concentration are real. This may be an artifact of the analysis. While the data suggests a

correlation, a clear biological explanation for this relationship is lacking.

Response 16: Because no effect is detected, a mechanistic explanation is no longer required, and the text now focuses on the SST signal alone.

Comments 17: Line 238: Authors did not find significant differences between protected and unprotected zones, and there are no visual indications of such differences. The text should avoid forcing the reader to perceive differences that do not exist.

Response 17: We have now found a significant relationship with the biomass, supporting these results.

Comments 18: Line 246 to 252: This is not entirely new information. The influence of UVC protocols on data collection is well-established. The authors need to provide a clearer explanation of their data collection methods to address potential biases.

Response 18: We agree that the influence of different UVC methodologies on fish density estimates is well-established in principle. However, our comparison specifically contrasts a "cross" protocol (two divers, cross-shaped transects) with a "comb" protocol (four or eight divers spaced laterally), a setup we have not found documented elsewhere. Because these methods differ in diver spacing and potential disturbance to fish, we believe it is valuable to quantify any bias between them. We have provided details in the Materials and Methods section on how each method was conducted and how we minimized potential biases (e.g., diver training, consistent transect layout, simultaneous observations recorded and then merged). If there are particular methodological clarifications the reviewer would recommend adding, we would be happy to elaborate further to ensure full transparency and address any remaining concerns.

Comments 19, Discussion: The discussion is overly lengthy and repetitive, delving into extensive literature reviews and explanations that are well-established in MPA science. Notably, the authors have omitted citing relevant studies that examined similar questions using the same species and yielded similar results. It remains unclear whether this oversight stems from a lack of thorough literature review or a deliberate decision to exclude these studies. I recommend restructuring the discussion to emphasize novel findings and comparisons with existing literature, providing a concise and clear overview. Furthermore, I suggest the authors incorporate recommendations to enhance the management of the studied MPA or to improve national MPA management efficiency as a way to integrate the new insights presented in the article.

Response 19: We have undertaken a full overhaul of the Discussion

Reviewer 2:

This study assessed the effectiveness of a no-take zone compared to two fished sites on Corsica. NTZ effectiveness was determined through UVCs of three predatory and threatened fish species. The study benefits from a relatively long temporal dataset, considerable diver time in conducting UVCs using two methods and across seasons and time of day, and good study design. The manuscript is very well written and effectively communicates the points of the authors. However, reviewing the manuscript left me with several unanswered questions. This manuscript could either benefit from including those explanations in the text and/or as a supplement or the authors could choose to address these questions directly to the reviewers.

Thank you for your positive feedback on the manuscript. We are pleased you found the study design robust and the manuscript clearly written. We have taken into account your comments and provided additional explanations in the revised text to address the questions you raised. We hope these clarifications meet your expectations, and we remain of course open to further suggestions.

Comments 20: Inclusion of environmental factors: Inclusion of environmental factors in the GLMs seems to have been an afterthought. I greatly appreciate the detailed explanation in the Discussion about the effect of the significant environmental factors on the population densities and distribution of the three species. But the Introduction does not provide adequate context as to why they were included in the first place. The authors could consider including a few sentences in the Introduction explaining why certain environmental

factors could influence fish population distributions. The inclusion of only significant environmental factors could, in a certain light, be viewed as cherry-picking or p-hacking. I encourage the authors to consider mentioning which other environmental factors were also analyzed and proved to be non-significant.

Response 20: We agree with the reviewer. We did not mean to suggest that including environmental variables was an afterthought, but rather a way to ensure that any reserve effects we observed were not confounded by environmental gradients. In the revised Introduction (Lines 8–22), we have clarified why temperature and chlorophyll-a were specifically chosen—namely, because temperature critically impacts fish physiology and distribution (e.g., growth, metabolism, spawning) while chlorophyll-a is a widely used proxy for primary productivity.

Comments 21: Study site effects vs fished and NTZ effects: The authors describe how one of the fished sites (Agriate) has a different habitat and bottom topography. Moreover, considerations of species size distributions are based upon site. Yet, in their presentation of the results the authors only present the effects of fished vs NTZ effects. I am curious as to what were the effects of individual sites on the GLMs? The manuscript would benefit greatly from a mention of whether this was tested and what the result was.

Response 21: As answered in response 12, we did, in fact, explore potential site-specific effects in preliminary analyses (Kruskal–Wallis, MANOVA, PERMANOVA, and initial GLMs incorporating site as a factor) to ensure differences were not driven primarily by habitat characteristics. These tests revealed no significant effect of regions (i.e., Nonza, Canelle and Agriate) on the densities of the species studied, aside from what was already captured by the “protected vs. unprotected” factor. Nonetheless, we have added a short note in the revised manuscript to clarify that we tested for site-level effects but found no statistically significant habitat-mediated differences that could overshadow protection status (Lines 214-220).

Comments 22: Study Protocol effects: Were divers in the Cross protocol trained using fish silhouette sizes before each season? If yes, please include a mention of this either by repeating the information or restructuring your description of the UVC protocols. Given the larger number of divers in the comb protocol vs cross protocol, did the authors consider the biases in fish size estimates and counts from differences in the number of observers? I appreciate the discussion of the pros and cons between these two methods. But the manuscript could benefit from a little more reflection on this or even potentially anecdotal mentions of inter-rater reliability if it was recorded during each season’s training session. Additionally, the authors could also reflect on the cost effectiveness of the cross vs comb technique, describing the differences in the amount of diver time as well as the area covered.

Response 22: We agree with the reviewer that the manuscript should include more reflection on this matter.

Diver Training for the Cross Protocol We confirm that divers conducting the cross protocol also participated in seasonal training sessions, using fish silhouette sizes to improve size estimation consistency—this was inadvertently omitted from our Methods description. We will revise the manuscript to clarify that both the cross and comb protocol divers received similar pre-season training with fish silhouettes (Lines 210-225)

Bias from Differences in the Number of Observers We acknowledge that having four or eight divers in the comb protocol could potentially introduce different biases compared to just two divers in the cross protocol. For instance, a larger team might increase the risk of double-counting a mobile fish, particularly if it swims along the transect line. However, comb protocol divers were instructed to maintain regular spacing (5–10 m) and communicate any sightings of highly mobile individuals to minimize double-counting. Additionally, after each dive, all observations were compiled and cross-checked to remove any potential duplicate entries. Our results showed no significant differences in density estimates for *D. dentex* between the two protocols, and for *S. umbra* and *E. marginatus*, densities were surprisingly lower with the comb approach—likely because multiple divers moving in parallel can frighten this territorial species into hiding. These findings suggest that any potential counting or sizing biases introduced by having more divers did not inflate density estimates.

Inter-Rater Reliability Although we did not systematically quantify inter-rater reliability after each training session, we did maintain consistent teams throughout the season whenever possible. Coupled with

the standardized training, we believe this approach minimized inter-observer discrepancies in size estimates and counts. In future studies, we plan to document inter-rater reliability more rigorously to provide quantitative insights into observer agreement.

Cost-Effectiveness and Logistical Considerations The comb protocol required substantially greater logistical effort, involving up to eight divers plus additional boat support for safety and coordination. Consequently, costs in terms of boat usage, fuel, and personnel time were higher than for the cross protocol (two divers plus a skipper). Organizing large teams and ensuring consistent availability of qualified divers also proved challenging. We initially hypothesized that the larger comb surveys would yield more accurate density estimates. However, given our findings (particularly for *E. marginatus*) and the associated costs, the cross protocol appears to be more cost-effective and less logistically demanding, especially when a reliable, well-trained diving team is available (Lines 554-562).

Comments 23: I commend the authors for their inclusion of descriptions of behaviours (eg. spawning aggregations, site fidelity, and nocturnal activity) affecting the distribution of fish species. The recommendations of adapting specific monitoring protocols to better estimate juveniles of populations or behavioral differences are also much appreciated.

Response 23: I sincerely thank the reviewer for these positive and encouraging comments, which are particularly motivating during the challenging process of writing the discussion.

Comments 24: Page 2, line 45: Consider replacing “fishing cantonnement” with “fishing area” to make it readable for a wider audience.

Response 24: To improve clarity for a wider audience while maintaining accuracy, we have now changed “fishing cantonnement” with “fishing exclusion zone.” I believe this term better reflects the no-fishing status of the area, as “fishing area” could be confusing given that fishing is prohibited there (Line 52).

Comments 25: Figures 3 and 4: Consider changing the color of the Cross Protocol bars and legend so that the lower limit of the black Standard Error bar is more visible.

Response 25: We appreciate the comment and adjusted the color to make the error bar more visible. The adjusted figures are attached to the revision.

Sincerely,
Dr. Lucie Vanalderweireldt
On behalf of all co-authors
